# Long-Term Cancer Outcomes Following Bariatric Surgery: A Comparative Analysis of Surgical Procedures

**DOI:** 10.3390/cancers16223730

**Published:** 2024-11-05

**Authors:** Jaewhan Kim, Kenechukwu C. Ben-Umeh, Joshua Kelley, Lance E. Davidson, Mia Hashibe, Ken Smith, Nathan Richards, Ted Adams

**Affiliations:** 1 Department of Physical Therapy, University of Utah, Salt Lake City, UT 84108, USA; joshua.kelley@utah.edu; 2 Department of Pharmacotherapy, University of Utah College of Pharmacy, Salt Lake City, UT 84112, USA; kenechukwu.ben-umeh@utah.edu; 3 Department of Exercise Sciences, Brigham Young University, Provo, UT 84602, USA; lance.davidson@byu.edu; 4 Division of Public Health, University of Utah, Salt Lake City, UT 84108, USA; mia.hashibe@utah.edu; 5 Department of Family Studies & Population Science, University of Utah, Salt Lake City, UT 84112, USA; ken.smith@fcs.utah.edu; 6 Intermountain Health, Murray, UT 84107, USA; nathan.richards@imail.org; 7 Division of Epidemiology, University of Utah, Salt Lake City, UT 84108, USA; ted.adams@utah.edu

**Keywords:** bariatric surgery, cancer incidence, Roux-en-Y gastric bypass, adjustable gastric banding, sleeve gastrectomy, duodenal switch

## Abstract

**Background/Objectives**: Metabolic and bariatric surgery (MBS) is known to reduce cancer risk. However, the association between specific bariatric procedures and cancer incidence is not well-studied. This study examined the association between four different MBS procedures and cancer incidence. **Methods**: Bariatric surgery registry data were linked with statewide cancer registry data from 1979 to 2018. The study included 27,092 adult subjects (aged ≥ 18 years old at surgery) who underwent MBS (BMI ≥ 30 kg/m^2^ at surgery) from 1979 to 2017. Cancer records were linked to MBS patient records, resulting in 1547 cancer cases. Cox proportional hazards regression was used to examine the association between MBS procedure types and cancer incidence. **Results**: Of all patients, 75% underwent Roux-en-Y gastric bypass (RYGB), 9% adjustable gastric banding (AGB), 10% sleeve gastrectomy (SG), and 6% duodenal switch (BPD-DS). The overall cancer incidence during the follow-up period was 6.4% for RYGB, 4.6% for AGB, 1.6% for SG, and 5.9% for BPD-DS. The mean follow-up duration from surgery to cancer incidence or censoring was 167 months (standard deviation = 121 months). Compared to RYGB, patients who underwent AGB (Hazard Ratio [HR] = 1.26, *p* = 0.03) and BPD-DS (HR = 1.91, *p* < 0.01) had a significantly higher hazard of developing cancer, while SG (HR = 1.17, *p* = 0.33) showed no significant difference. **Conclusions**: These findings suggest that AGB and BPD-DS may be associated with higher cancer risks compared to RYGB. Additional large population studies are needed to better understand the long-term cancer risks and mechanisms associated with different MBS types.

## Simple Summary

This study examined the link between different metabolic and bariatric surgery (MBS) procedures and cancer incidence by linking the statewide bariatric surgery registry with the cancer registry (1979–2018) for 27,092 adult patients. Four types of surgeries were compared: Roux-en-Y gastric bypass (RYGB), adjustable gastric banding (AGB), sleeve gastrectomy (SG), and biliopancreatic diversion with duodenal switch (BPD-DS). Over a follow-up period averaging 167 months, cancer incidence was 6.4% for RYGB, 4.6% for AGB, 1.6% for SG, and 5.9% for BPD-DS. We found that patients who underwent AGB and BPD-DS were more likely to develop cancer compared to those who had RYGB. These findings highlight that cancer hazards differ by MBS procedure, and there may be the need for further research with more data to better understand the differences observed in our study.

## 1. Introduction

Metabolic and bariatric surgery (MBS) is frequently used as an effective long-term treatment for individuals with severe obesity [1]. There are multiple MBS procedures, each of which results in differing weight loss, diabetes remission, and other health outcomes [2,3,4]. Laparoscopic adjustable gastric banding (AGB) and sleeve gastrectomy (SG) are two common surgeries that restrict food intake, whereas Roux-en-Y gastric bypass (RYGB) and biliopancreatic diversion with duodenal switch (BPD-DS) are MBS procedures that combine both restrictive and malabsorptive components [2,5,6].

The impact of MBS on cancer risk remains controversial. Some studies have reported an increased risk of colorectal and kidney cancers following MBS [7,8], while other studies have found that MBS patients, when matched to non-surgery patients with severe obesity, experience a 25–50% reduction in cancer risk [8,9,10,11,12]. Additionally, cancer-related deaths following RYGB surgery have been shown to decrease by 46–60% compared to matched non-surgery subjects with severe obesity [11,13]. Furthermore, studies have reported that following MBS the incidence of obesity-related cancers, such as endometrial cancer and postmenopausal breast cancer, decreases more significantly than that of non-obesity-related cancers [7,8,10,14].

Previous findings suggest that RYGB is associated with the most significant reduction in the risk of hormone-related cancers compared to SG and AGB. Nonetheless, both SG and AGB are also linked to a substantial reduction in cancer risk when compared to a non-surgical, obesity-matched cohort [7]. RYGB has been associated with a lower hazard of developing female-specific cancers compared to SG and AGB [15]. However, some research suggests that the hazard rates for cancer may be similar across these surgical procedures [9,16]. Additionally, studies have compared cancer-specific incidences by surgery type for colorectal, gastrointestinal, and esophageal cancers. Both SG and RYGB were shown to have a lower incidence of colorectal cancer and benign polyps compared to AGB [17]. Recent case-control studies have also examined gastric and esophageal cancer risks following AGB, SG, and BPD-DS [18,19]. Both AGB and SG are thought to increase the risk of esophageal cancer due to the high cumulative incidence of Gastroesophageal Reflux Disease (GERD) compared to RYGB [20,21]. However, there are limited data to conclude whether the risk of developing esophageal cancer differs among these surgical procedures [22].

While there is speculation that cancer risk may vary depending on the type of bariatric surgery performed [23], the relationship between specific procedures and cancer incidence has not been extensively studied. Previous studies have relatively short follow-up periods with limited sample sizes and do not include large numbers of patients for each specific MBS procedure type. Accessing data from 1979 to 2018, this paper examined the association between four different MBS procedures and cancer incidence.

## 2. Materials and Methods

### 2.1. Subjects

Adult subjects (≥18 years old at surgery) who underwent bariatric surgery between 1979 and 2017 were selected. Patients who had undergone RYGB, AGB, SG, or BPD-DS were included in the study. All patients had a body mass index (BMI) ≥30 kg/m^2^ at surgery. Patients who had any type of cancer diagnosed before or at the time of bariatric surgery were excluded, as well as those with missing data on surgery type. This study was approved by the University of Utah Institutional Review Board (IRB_00095902).

### 2.2. Data

This study utilized three statewide datasets: the Utah Bariatric Surgery Registry (UBSR), the Utah Cancer Registry (UCR), a part of the Surveillance, Epidemiology, and End Results (SEER) program, and the Utah Population Database (UPDB). The UBSR included information on patients who had undergone bariatric surgery from 1979 to 2017. For each patient, the registry included data on height, weight, BMI at surgery, type 2 diabetes status, age at surgery, sex, race/ethnicity, and the type of bariatric surgery performed. The UCR provided statewide cancer statistics, including all cancer records in the state since 1966 [24]. The UCR included cancer diagnosis codes based on ICD-O-3 site, cancer stage at diagnosis, ICD-O-3 histology code, cancer behavior code, histology grade, diagnosis date, and SEER code [24]. The UPDB, a comprehensive statewide database of linked records [25], provided detailed demographic information, including birth date, death date, date last seen alive in Utah, race/ethnicity, and marital status. The study linked the UBSR with the UCR to identify any cancer incidence among surgical subjects between 1979 and 2018. The UPDB was also used to identify death date and last living date (censoring date) in Utah for the analysis.

### 2.3. Outcome

The UBSR data were linked to the UCR to identify cancer incidence, using the date of cancer diagnosis following MBS. Cancer identification was based on ICD-O-3 (type and histology) codes and SEER codes [26]. The study included all stages of first incident cancers in subjects who underwent MBS: carcinoma in situ, localized, regional, distance metastases/systemic disease, and unknown stages. The types of cancer examined included those affecting the oral cavity and pharynx, digestive system, respiratory system, bones and joints, soft tissue, skin, breast, genital system (both male and female), urinary system, eye and orbit, brain and nervous system, endocrine, lymphoma, myeloma, leukemia, mesothelioma, Kaposi sarcoma, and miscellaneous cancers. The earliest diagnosed cancer was recorded as the outcome and was defined as a binary variable (Yes or No). In addition, cancer types were categorized into obesity-related and non-obesity-related cancers. Obesity-related cancers included esophageal, breast (in postmenopausal women aged ≥55 years), colorectal, uterine, gallbladder, stomach, kidney, liver, ovarian, pancreatic, thyroid cancers, as well as meningioma and myeloma [27,28]. Non-obesity-related cancers included all other cancer types.

### 2.4. Type of Surgery

The primary independent variable in this study was the type of MBS procedure, as identified from the UBSR. The registry provided Current Procedure Terminology (CPT) and International Classification of Disease-9/10 procedure codes (ICD-9/10 PCS) [29]. Based on these codes, the procedures were classified into RYGB, AGB, SG, and BPD-DS.

### 2.5. Covariates

In addition to the type of surgery, age at surgery (in years), sex (male, female), race/ethnicity (non-Hispanic White, Hispanic, and non-Hispanic non-White), BMI category (30–34.9, 35–39.9, 40–44.9, 45–49.9, and ≥50 kg/m^2^) at surgery, and rural/urban residence at surgery were adjusted for in the analysis. Rural–Urban Commuting Area (RUCA) codes were used to identify rural vs. urban residents. RUCA codes 1.0–3.0, 4.1, 5.1, 7.1, 8.1. and 10.1 were defined as urban, while others codes were defined as rural [30]. Type 2 diabetes status prior to or at the time of surgery was also recorded. These baseline characteristics were from the UBSR, while the rural/urban residence data were from the UPDB.

### 2.6. Statistical Approach

Summary statistics such as mean, standard deviations (SD), and percentages were used to summarize the baseline characteristics of the subjects. For comparisons of baseline characteristics and cancer incidence across different types of procedures, multiple comparisons were conducted using analysis of variance (ANOVA) with the Šidák method to adjust *p*-values for continuous variables, while chi-square tests were used to compare categorical variables. Cox proportional hazards regression was used to assess the association between procedure type and cancer incidence, controlling for baseline characteristics. An event was defined as the first cancer incidence following surgery. Censoring occurred under the following conditions: (1) when subjects relocated (with the last known date of residence in Utah as the censoring date); (2) upon death; or (3) on 31 December 2018, for patients living in Utah. The proportional hazards (PH) assumption was tested with scaled Schoenfeld residuals. The assumption was found to be violated for overall cancer incidence (*p*-value = 0.03) due to the age variable. Corrections were made by introducing an interaction term between time and the age variable that violated the PH assumption to accurately estimate the hazard ratios for the covariates [31,32]. A *p*-value less than or equal to 0.05 was considered as statistically significant. Stata (version 18.0, College Station, Texas) was used for the analyses in the study.

## 3. Results

A total of 28,258 subjects underwent bariatric surgery between 1979 and 2017. Of these, subjects with missing surgery type (n = 819) and those with a BMI less than 30 kg/m^2^ (n = 347) were excluded from the analysis. The final sample size was 27,092 subjects of whom 1547 (5.74%) were diagnosed with cancer following bariatric surgery. Table 1 presents the characteristics of patients across the different procedure types. Mean (SD) follow-up time from surgery to cancer incidence or censoring was 167 (121) months, or about 14 years. The mean (SD) durations from surgery to follow-up were 168 (120) months for RYGB, 201 (120) months for AGB, 104 (43) months for SG, and 81 (64) months for BPD-DS. Among surgical patients, 75%, 9%, 10%, and 6% underwent RYGB, GB, SG, and BPD-DS, respectively. The overall average (SD) age at surgery was 42 (11) years, 80% of patients were female, and 73% were non-Hispanic White. The average (SD) BMI at surgery was 45.5 (8.3) kg/m^2^.

Cancer incidence following MBS was 6.4% for RYGB, 4.6% for AGB, 1.6% for SG, and 5.9% for BPD-DS. The incidence of obesity-related cancers by procedure type was 2.9% for RYGB, 2.0% for AGB, 0.7% for SG, and 3.3% for BPD-DS. Non-obesity-related cancer incidence by procedure type was 3.5% for RYGB, 2.5% for AGB, 0.9% for SG, and 2.6% for BPD-DS.

Cox regression showed that subjects who underwent AGB (HR = 1.26, *p* = 0.03) and BPD-DS (HR = 1.91, *p* < 0.001) had higher hazards of developing cancer compared to those who underwent RYGB (Table 2). Patients who underwent SG (HR = 1.17, *p* = 0.33) did not have a statistically significant difference in cancer hazard compared to those who underwent RYGB. However, patients with a diabetes diagnosis at baseline had a higher hazard of developing cancer compared to those without diabetes (HR = 1.38, *p* < 0.001). Female patients had a lower hazard of cancer than male patients (HR = 0.79, *p* < 0.001), and those with a BMI of 40–44.9 kg/m^2^ at the time of surgery had a lower cancer hazard compared to those with a BMI ≥50 kg/m^2^ (HR = 0.85, *p* = 0.02).

After controlling for covariates, Cox regression showed that subjects who underwent BPD-DS (HR = 2.02, *p* < 0.001) had a higher hazard of obesity-related cancer incidence compared to those who underwent RYGB, while both AGB and SG did not show significantly different hazards compared to RYGB (Table 3). Patients with a lower BMI at the time of surgery had a reduced hazard of cancer compared to those with a BMI ≥50 kg/m^2^ (35–39.9 kg/m^2^: HR = 0.71, *p* < 0.001; 40–44.9 kg/m^2^: HR = 0.70, *p* < 0.01, 45–49.9 kg/m^2^: HR = 0.71, *p* < 0.001). However, in contrast to overall cancer incidence, female patients had a higher hazard of obesity-related cancers compared to male patients (HR = 1.45, *p* < 0.001).

For non-obesity-related cancers, subjects who underwent BPD-DS (HR = 1.61, *p* < 0.001) had a higher hazard of incidence compared to those who underwent RYGB (Table 4). No significant differences were found between RYGB and AGB (HR = 1.29, *p* = 0.06), or between RYGB and SG (HR = 1.27, *p* = 0.28). Additionally, no significant differences were observed by BMI categories. However, female patients had a significantly lower hazard of non-obesity-related cancers (HR = 0.54, *p* < 0.001).

## 4. Discussion

This study accessed statewide bariatric surgery and cancer registries to capture the incidence of cancer following bariatric surgery, with a long-term follow-up of patients (mean 14 years; maximum 38 years). By examining cancer incidence and the time from MBS to cancer diagnosis, this study showed differences in cancer incidence by procedure type. Patients who underwent BPD-DS had a 1.91-times-higher risk, and those who underwent AGB had a 1.26-times-higher risk for all-cause cancer incidence compared to RYGB, while SG showed no significant difference in cancer risk relative to RYGB. Additionally, BPD-DS was associated with a significantly higher risk of both obesity-related cancers (HR = 2.02) and non-obesity-related cancers (HR = 1.61).

Although many studies have explored the relationship between cancer and MBS, few have compared cancer risks by surgical procedure. Mackenzie et al. and Tsui et al. reported differences in cancer incidence by bariatric surgery type [7,15]. Tsui et al. found a 34% decrease in hazard for female-specific cancers for RYGB compared to SG, and a 17% decrease compared to AGB [15]. Mackenzie et al. reported a greater reduction in the risk of hormone-related cancers for RYGB (OR = 0.16) compared to controls, while AGB and SG had ORs of 0.34 and 0.21, respectively, compared to controls. Over a 15-year period (mean follow-up of 4.6 years), cumulative incidence differed by surgery type—1.8% for RYGB (90/4978), 1.4% for SG (12/859), and 3.4% for AGB (101/2957) [7]. However, no direct statistical comparisons were made between the procedures. Another matched-cohort study reported adjusted HR of 0.70 for RYGB and 0.66 for SG compared to the non-surgical control group [33]. Two additional studies with similar findings reported hazard ratios and incidence ratios for cancer by surgery type. Sjostrom et al. reported HRs of 0.54 for both RYGB and AGB in women compared to a control group with obesity [9]. Ostlund et al. reported similar standardized cancer incidence rates between patients that underwent AGB (SIR = 1.05) and RYGB (SIR = 1.01) compared to the general Swedish population [16].

In terms of cancer-specific outcomes, Bailly et al. reported lower cumulative incidences of colorectal cancer for SG and RYGB (0.5% each) compared to AGB (0.7%), with higher rates of benign polyps in AGB (5.0%) compared to both SG and RYGB (3.1% each) [17]. AGB and SG are thought to increase the risk of esophageal cancer due to a higher prevalence of GERD [20,34]. Abu Sneineh et al. reported GERD rates of 39.9% for SG, 23.4% for AGB, and 11% for RYGB [35]. However, data on whether esophageal cancer risk varies by procedure type remain limited. A recent meta-analysis found no significant association between bariatric surgery and esophageal cancer incidence, but it did not delve into differentiating between procedure types [36]. Andalib et al. attempted to compare procedures but, with only eight esophageal cancer cases in 4874 participants over 7.6 years, the study was underpowered to draw conclusions [22].

The link between cancer and obesity is believed to stem from changes in the endocrine, inflammatory, and immune systems [8]. Adipose tissue, a major endocrine organ, produces high levels of estrogen and adipokines [37], both linked to an increased risk of breast, endometrial, and ovarian cancers [38,39]. Furthermore, leptin, a pro-inflammatory adipokine, may drive cell proliferation and influence cell growth through pathways such as mTOR and AMP-activated protein kinase [40]. MBS induces metabolic changes, including changes in sex hormones, gut hormones, cellular energetics, and immune response [41,42], all of which may lead to a decreased risk of hormone-related cancers [7,23]. These physiologic and biochemical changes in the body vary by surgical procedure and may positively or negatively impact cancer risk [13,19].

Differences in diabetes remission and insulin levels from various bariatric procedures may explain variations in cancer risk. Research has established an association between diabetes and increased cancer incidence, with elevated insulin and insulin-like growth factor 1 (IGF-1) levels in diabetes and obesity promoting cell growth [41,43]. Lower post-MBS insulin and IGF-1 levels likely reduce cancer risk [23]. Metabolic responses vary by procedure; RYGB and SG are more effective than AGB in achieving weight loss and T2DM remission [44], though differences in outcomes between RYGB and SG remain controversial. Some studies suggest that the two procedures result in relatively similar rates of weight loss and T2DM remissions [44,45], while others indicate that RYGB provides more durable T2DM remission and glycemic control [3,46]. BPD-DS, though less common, typically leads to the most substantial weight loss and T2DM remission rate and is often used to treat very severe obesity [2]. Post-surgical weight loss varies between procedures [4,44,47], but the extent to which weight loss alone influences cancer incidence, independent of surgical procedure, remains controversial. Schauer et al. demonstrated that for every 10% of weight loss there was an associated HR of 0.89 for cancer risk [48]. In contrast, Sjostrom et al. found no significant association between weight loss and reduced cancer incidence [9]. Additionally, baseline differences in weight, diabetes status, and insulin resistance are seen across different surgical procedures [4] and may be confounding factors. Our analysis showed that patients who underwent BPD-DS had an increased cancer risk even after controlling for T2DM and BMI, suggesting that additional factors may contribute to the increased cancer risk associated with BPD-DS.

This study followed subjects who underwent MBS for an average of 14 years and included BPD-DS, RYGB, GB, and SG. Despite its strengths, several limitations could affect the results. First, baseline comorbidities such as mental health conditions were not considered. Second, clinical values such as BMI and A1c changes following surgery were not assessed. They could be confounding variables but were not considered in the study. Third, information on prescriptions, nutritional disorders, smoking, and alcohol consumption were not adjusted for in the regression. Finally, patients who underwent MBS revision surgery were analyzed based upon their initial surgery and the revision was not taken into consideration for the analysis. Despite several limitations, this study captures extensive follow-up data on the most common bariatric surgical procedures in Utah and presents different cancer risks associated with these procedure types.

## 5. Conclusions

Our findings suggest that AGB and BPD-DS may be associated with higher cancer risks compared to RYGB. Research on cancer risk associated with different bariatric surgery procedures remains controversial, and the underlying mechanisms for risk reduction or promotion are poorly understood. Long-term follow-up and comprehensive data collection are essential for accurately assessing cancer incidence after MBS. The observed differences in cancer incidence—both for obesity-related and non-obesity-related cancers—linked to specific surgical types in our study highlights the need for further research that includes extended follow-up and detailed data collection post-MBS.

## Figures and Tables

**Table 1:** Baseline characteristics of subjects at the time of surgery by procedure type.

	Total	RYGB	AGB	SG	BPD-DS	*p*-Value
	N = 27,092; 100%	N = 20,220; 74.63%	N = 2546; 9.40%	N = 2761; 10.19%	N = 1565; 5.78%	
	Mean (SD)/%	Mean (SD)/%	Mean (SD)/%	Mean (SD)/%	Mean (SD)/%	
Follow-up months	166.7 (121.1)	198.6 (121.5)	103.1 (43.9)	41.8 (25.5)	78.5 (62.8)	<0.001
Cancer occurrence (%)						
All cancer	5.7	6.4	4.6	1.6	5.9	<0.001
Obesity-related cancers	2.6	2.9	2.0	0.7	3.3	<0.001
Non-obesity-related cancers	3.1	3.5	2.5	0.9	2.6	<0.001
Person–year incidence rate (per 1000 person–years)						
All cancers (95% CI)	4.07 (3.87–4.28)	3.83 (3.62–4.05)	5.31 (4.42–6.36)	4.48 (3.32–6.03)	8.70 (7.01–10.71)	<0.001
Obesity-related cancers (95% CI)	1.85 (1.71–1.99)	1.72 (1.58–1.87)	2.38 (1.81–3.12)	1.98 (1.26–3.10)	4.79 (3.62–6.34)	<0.001
Non-obesity-related cancers (95% CI)	2.22 (2.07–2.37)	2.11 (1.96–2.28)	2.93 (2.89–3.74)	2.50 (1.67–3.72)	3.91 (2.87–5.33)	<0.001
Age	41.8 (11.4)	41.1 (11.1)	43.3 (12.1)	44.0 (11.5)	44.9 (12.6)	<0.001
Female (%)	80.3	82.1	78.2	74.0	71.4	<0.001
Race/ethnicity (%)						<0.001
Non-Hispanic White	72.6	71.5	79.9	73.0	74.2	
Non-Hispanic others *	9.1	10.8	4.2	5.0	3.3	
Hispanic	18.3	17.7	15.8	22.1	22.6	
Residence (%)						<0.001
Rural	11.8	13.7	19.0	10.6	12.6	
Urban	72.5	70.9	82.1	70.4	80.8	
Unknown	14.9	17.4	4.3	10.5	8.8	
BMI category (%)						<0.001
30–34.9	3.3	2.3	9.2	3.7	5.7	
35–39.9	21.0	20.6	26.2	21.9	15.8	
40–44.9	30.5	31.0	29.8	32.2	21.1	
45–49.9	21.0	21.5	19.1	20.0	18.8	
≥50	24.2	24.5	15.7	22.2	38.6	
Diabetes (%)	18.6	16.2	19.8	27.5	32.7	<0.001

RYGB: Roux-en-Y gastric bypass; AGB: adjustable gastric banding; SG: sleeve gastrectomy; BPD-DS: biliopancreatic diversion with duodenal switch; *: non-Hispanic others include African American, American Indian or Alaska Native, Native Hawaiian or Other Pacific Islander, and Asian.

**Table 2:** Factors associated with overall cancer incidence following bariatric surgery.

Covariates	Hazard Ratio	*p*-Value	95% CI	
Procedure type				
RYGB	Reference			
AGB	1.26	0.03	1.03	1.53
SG	1.17	0.33	0.85	1.61
BPD-DS	1.91	<0.001	1.53	2.39
Age at surgery	1.01	<0.001	1.01	1.01
Female	0.79	<0.001	0.69	0.89
Race/ethnicity				
Non-Hispanic White	Reference			
Hispanic	0.89	0.09	0.77	1.02
Non-Hispanic others	0.15	<0.001	0.09	0.26
Residence				
Urban	Reference			
Rural	1.10	0.18	0.95	1.28
Unknown	0.33	<0.001	0.25	0.44
BMI category				
30–34.9	0.82	0.18	0.61	1.10
35–39.9	0.87	0.06	0.74	1.01
40–44.9	0.85	0.02	0.73	0.97
45–49.9	0.88	0.11	0.75	1.03
≥50	Reference			
Diabetes	1.38	<0.001	1.20	1.60

**Table 3:** Factors associated with obesity-related cancer incidence following bariatric surgery.

Covariate	HR	*p*-Value	95% CI	
Procedure type				
RYGB	Reference			
AGB	1.14	0.40	0.85	1.53
SG	0.98	0.94	0.61	1.58
BPD-DS	2.02	<0.001	1.49	2.74
Age at surgery	1.08	<0.001	1.07	1.08
Female	1.45	<0.001	1.17	1.81
Race/ethnicity				
Non-Hispanic White	Reference			
Hispanic	0.94	0.57	0.77	1.15
Non-Hispanic others	0.24	<0.001	0.13	0.46
Residence				
Urban	Reference			
Rural	1.24	0.06	0.99	1.55
Unknown	0.37	<0.001	0.25	0.56
BMI category				
30–34.9	0.70	0.09	0.46	1.06
35–39.9	0.71	<0.001	0.57	0.88
40–44.9	0.70	<0.001	0.56	0.89
45–49.9	0.71	<0.001	0.57	0.87
≥50	Reference			
Diabetes	1.14	0.24	0.92	1.41

**Table 4:** Factors associated with non-obesity-related cancer incidence following bariatric surgery.

Covariate	HR	*p*-Value	95% CI	
Procedure type				
RYGB	Reference			
AGB	1.29	0.06	0.99	1.69
SG	1.27	0.28	0.83	1.94
BPD-DS	1.61	<0.001	1.16	2.24
Age at surgery	1.05	<0.001	1.05	1.06
Female	0.54	<0.001	0.46	0.63
Race/ethnicity				
Non-Hispanic White	Reference			
Hispanic	0.85	0.09	0.71	1.03
Non-Hispanic others	0.09	<0.001	0.04	0.22
Residence				
Urban	Reference			
Rural	1.03	0.77	0.85	1.25
Unknown	0.31	<0.001	0.21	0.45
BMI category				
30–34.9	0.91	0.66	0.60	1.38
35–39.9	1.02	0.87	0.82	1.26
40–44.9	0.98	0.83	0.80	1.19
45–49.9	1.05	0.64	0.85	1.30
≥50	Reference			
Diabetes	1.39	<0.001	1.13	1.69

## Data Availability

Restrictions apply to the availability of these data. Data cannot be shared publicly because of the regulations of the Utah Department of Health and Human Services, and the Utah Resource for Genetic and Epidemiologic Research (RGE). Data are available from the University of Utah Institutional Data Access/Ethics Committee (contact via RGE) for researchers who meet the criteria for access to confidential data.

## References

[1] Golzarand M., Toolabi K., Farid R. (2017). The bariatric surgery and weight losing: A meta-analysis in the long- and very long-term effects of laparoscopic adjustable gastric banding, laparoscopic Roux-en-Y gastric bypass and laparoscopic sleeve gastrectomy on weight loss in adults. Surg. Endosc..

[2] Sudlow A., le Roux C.W., Pournaras D.J. (2020). The metabolic benefits of different bariatric operations: What procedure to choose?. Endocr. Connect..

[3] McTigue K.M., Wellman R., Nauman E., Anau J., Coley R.Y., Odor A., Tice J., Coleman K.J., Courcoulas A., Pardee R.E. (2020). Comparing the 5-Year Diabetes Outcomes of Sleeve Gastrectomy and Gastric Bypass: The National Patient-Centered Clinical Research Network (PCORNet) Bariatric Study. JAMA Surg..

[4] Courcoulas A.P., Christian N.J., Belle S.H., Berk P.D., Flum D.R., Garcia L., Horlick M., Kalarchian M.A., King W.C., Mitchell J.E. (2013). Weight change and health outcomes at 3 years after bariatric surgery among individuals with severe obesity. JAMA.

[5] Bruno D.S., Berger N.A. (2020). Impact of bariatric surgery on cancer risk reduction. Ann. Transl. Med..

[6] Gagnon C., Schafer A.L. (2018). Bone Health After Bariatric Surgery. JBMR Plus.

[7] Mackenzie H., Markar S.R., Askari A., Faiz O., Hull M., Purkayastha S., Moller H., Lagergren J. (2018). Obesity surgery and risk of cancer. Br. J. Surg..

[8] Tao W., Santoni G., von Euler-Chelpin M., Ljung R., Lynge E., Pukkala E., Ness-Jensen E., Romundstad P., Tryggvadottir L., Lagergren J. (2020). Cancer Risk After Bariatric Surgery in a Cohort Study from the Five Nordic Countries. Obes. Surg..

[9] Sjostrom L., Gummesson A., Sjostrom C.D., Narbro K., Peltonen M., Wedel H., Bengtsson C., Bouchard C., Carlsson B., Dahlgren S. (2009). Effects of bariatric surgery on cancer incidence in obese patients in Sweden (Swedish Obese Subjects Study): A prospective, controlled intervention trial. Lancet Oncol..

[10] Schauer D.P., Feigelson H.S., Koebnick C., Caan B., Weinmann S., Leonard A.C., Powers J.D., Yenumula P.R., Arterburn D.E. (2019). Bariatric Surgery and the Risk of Cancer in a Large Multisite Cohort. Ann. Surg..

[11] Adams T.D., Stroup A.M., Gress R.E., Adams K.F., Calle E.E., Smith S.C., Halverson R.C., Simper S.C., Hopkins P.N., Hunt S.C. (2009). Cancer incidence and mortality after gastric bypass surgery. Obesity.

[12] Adams T.D., Meeks H., Fraser A., Davidson L.E., Holmen J., Newman M., Ibele A.R., Playdon M., Hardikar S., Richards N. (2023). Long-term cancer outcomes after bariatric surgery. Obesity.

[13] Adams T.D., Hunt S.C. (2009). Cancer and obesity: Effect of bariatric surgery. World J. Surg..

[14] Ishihara B.P., Farah D., Fonseca M.C.M., Nazario A. (2020). The risk of developing breast, ovarian, and endometrial cancer in obese women submitted to bariatric surgery: A meta-analysis. Surg. Obes. Relat. Dis..

[15] Tsui S.T., Yang J., Zhang X., Spaniolas K., Kim S., Griffin T., Burke W.M., Pryor A.D. (2021). The risk of female-specific cancer after bariatric surgery in the state of New York. Surg. Endosc..

[16] Ostlund M.P., Lu Y., Lagergren J. (2010). Risk of obesity-related cancer after obesity surgery in a population-based cohort study. Ann. Surg..

[17] Bailly L., Fabre R., Pradier C., Iannelli A. (2020). Colorectal Cancer Risk Following Bariatric Surgery in a Nationwide Study of French Individuals With Obesity. JAMA Surg..

[18] Angrisani L., Palma R., Santonicola A., Ferraro L., Iovino P. (2020). Sleeve Gastrectomy and Gastric Cancer: Is It Really Rare?. Obes. Surg..

[19] Ebrahimi R., Kermansaravi M., Khalaj A., Eghbali F., Mousavi A., Pazouki A. (2019). Gastro-Intestinal Tract Cancers Following Bariatric Surgery: A Narrative Review. Obes. Surg..

[20] Gehwolf P., Kienzl-Wagner K., Cakar-Beck F., Schafer A., Wykypiel H. (2019). Laparoscopic Adjustable Gastric Banding: An Underestimated Risk Factor for the Development of Esophageal Cancer?-a Nationwide Survey. Obes. Surg..

[21] Plat V.D., Kasteleijn A., Greve J.W.M., Luyer M.D.P., Gisbertz S.S., Demirkiran A., Daams F. (2021). Esophageal Cancer After Bariatric Surgery: Increasing Prevalence and Treatment Strategies. Obes. Surg..

[22] Andalib A., Bouchard P., Demyttenaere S., Ferri L.E., Court O. (2021). Esophageal cancer after sleeve gastrectomy: A population-based comparative cohort study. Surg. Obes. Relat. Dis..

[23] Sauter E.R., Heckman-Stoddard B. (2021). Metabolic Surgery and Cancer Risk: An Opportunity for Mechanistic Research. Cancers.

[24] Utah Population Database. https://uofuhealth.utah.edu/huntsman/utah-population-database/data/.

[25] Smith K.R., Mineau G.P. (2021). The Utah Population Database. The Legacy of Four Decades of Demographic Research. Hist. Life Course Stud..

[26] Site Recode ICD-0-3/WHO 2008 Definition: National Cancer Institute. https://seer.cancer.gov/siterecode/icdo3_dwhoheme/index.html.

[27] Does Body Weight Affect Cancer Risk?: American Cancer Society. https://www.cancer.org/cancer/cancer-causes/diet-physical-activity/body-weight-and-cancer-risk/effects.html.

[28] Obesity and Cancer: Division of Cancer Prevention and Control, Centers for Disease Control and Prevention. https://www.cdc.gov/cancer/obesity/index.htm.

[29] Kim J., Waitzman N., Simper S., McKinlay R., Cottam D., Surve A., Richards N., Adams T. (2021). Effects of Post-operative Nutritional Disorders Following Bariatric Surgery on Health Care Cost and Use. Obes. Surg..

[30] Rural Health Research Center Data Codes. https://depts.washington.edu/uwruca/ruca-uses.php.

[31] Jin S., Boehmke F.J. (2017). Proper Specification of Nonproportional Hazards Corrections in Duration Models. Political Anal..

[32] Howard R., Chao G.F., Yang J., Thumma J., Chhabra K., Arterburn D.E., Ryan A., Telem D.A., Dimick J.B. (2021). Comparative Safety of Sleeve Gastrectomy and Gastric Bypass Up to 5 Years After Surgery in Patients With Severe Obesity. JAMA Surg..

[33] Aminian A., Wilson R., Al-Kurd A., Tu C., Milinovich A., Kroh M., Rosenthal R.J., Brethauer S.A., Schauer P.R., Kattan M.W. (2022). Association of Bariatric Surgery With Cancer Risk and Mortality in Adults With Obesity. JAMA.

[34] Lazzati A., Poghosyan T., Touati M., Collet D., Gronnier C. (2023). Risk of Esophageal and Gastric Cancer After Bariatric Surgery. JAMA Surg..

[35] Abu Sneineh M., Abu Sneineh M., Abu Sneineh M., Abu Sneineh M., Abu Sneineh M., Abu Sneineh M. (2021). Sleeve Gastrectomy Is the Most Common Cause of Gastroesophageal Reflux Disease in Comparison with Other Bariatric Operations. Dig. Dis..

[36] Wilson R.B., Lathigara D., Kaushal D. (2023). Systematic Review and Meta-Analysis of the Impact of Bariatric Surgery on Future Cancer Risk. Int. J. Mol. Sci..

[37] Coelho M., Oliveira T., Fernandes R. (2013). Biochemistry of adipose tissue: An endocrine organ. Arch. Med. Sci..

[38] Zhang X., Tworoger S.S., Eliassen A.H., Hankinson S.E. (2013). Postmenopausal plasma sex hormone levels and breast cancer risk over 20 years of follow-up. Breast Cancer Res. Treat..

[39] Farhat G.N., Parimi N., Chlebowski R.T., Manson J.E., Anderson G., Huang A.J., Vittinghoff E., Lee J.S., Lacroix A.Z., Cauley J.A. (2013). Sex hormone levels and risk of breast cancer with estrogen plus progestin. J. Natl. Cancer Inst..

[40] Kolb R., Sutterwala F.S., Zhang W. (2016). Obesity and cancer: Inflammation bridges the two. Curr. Opin. Pharmacol..

[41] Ashrafian H., Ahmed K., Rowland S.P., Patel V.M., Gooderham N.J., Holmes E., Darzi A., Athanasiou T. (2011). Metabolic surgery and cancer: Protective effects of bariatric procedures. Cancer.

[42] Casimiro I., Sam S., Brady M.J. (2019). Endocrine implications of bariatric surgery: A review on the intersection between incretins, bone, and sex hormones. Physiol. Rep..

[43] Gallagher E.J., LeRoith D. (2015). Obesity and Diabetes: The Increased Risk of Cancer and Cancer-Related Mortality. Physiol. Rev..

[44] Pham S., Gancel A., Scotte M., Houivet E., Huet E., Lefebvre H., Kuhn J.M., Prevost G. (2014). Comparison of the effectiveness of four bariatric surgery procedures in obese patients with type 2 diabetes: A retrospective study. J. Obes..

[45] Lee Y., Doumouras A.G., Yu J., Aditya I., Gmora S., Anvari M., Hong D. (2021). Laparoscopic Sleeve Gastrectomy Versus Laparoscopic Roux-en-Y Gastric Bypass: A Systematic Review and Meta-analysis of Weight Loss, Comorbidities, and Biochemical Outcomes From Randomized Controlled Trials. Ann. Surg..

[46] Wood M.H., Carlin A.M., Ghaferi A.A., Varban O.A., Hawasli A., Bonham A.J., Birkmeyer N.J., Finks J.F. (2019). Association of Race With Bariatric Surgery Outcomes. JAMA Surg..

[47] Strain G.W., Gagner M., Pomp A., Dakin G., Inabnet W.B., Hsieh J., Heacock L., Christos P. (2009). Comparison of weight loss and body composition changes with four surgical procedures. Surg. Obes. Relat. Dis..

[48] Schauer D.P., Feigelson H.S., Koebnick C., Caan B., Weinmann S., Leonard A.C., Powers J.D., Yenumula P.R., Arterburn D.E. (2017). Association Between Weight Loss and the Risk of Cancer after Bariatric Surgery. Obesity.

